# Stress evaluation of different cad-on material combinations in crown design over conventional hand-layered zirconia - an in vitro 3d finite element analysis

**DOI:** 10.21142/2523-2754-1401-2026-270

**Published:** 2025-12-28

**Authors:** Mehtab Singh Sidhu, Kiran Kumar Pandurangan, Subhabrata Maiti, Delphine Priscilla Antony, Klara Avetisyan, Artak Heboyan

**Affiliations:** 1 Department of Prosthodontics, Saveetha Dental College and Hospitals, Saveetha Institute of Medical and Technical Sciences, Saveetha University. Chennai, Tamil Nadu, India. mehtabssidhu1@gmail.com dr.kirankumar789@gmail.com drsubhoprostho@gmail.com Department of Prosthodontics Saveetha Dental College and Hospitals Saveetha Institute of Medical and Technical Sciences, Saveetha University Chennai, Tamil Nadu India mehtabssidhu1@gmail.com dr.kirankumar789@gmail.com drsubhoprostho@gmail.com; 2 Department of Conservative and Endodontics, Saveetha Dental College and Hospitals, Saveetha Institute of Medical and Technical Sciences, Saveetha University. Chennai, Tamil Nadu, India. delphine.sdc@saveetha.com Department of Conservative and Endodontics College and Hospitals Saveetha Institute of Medical and Technical Sciences, Saveetha University Chennai, Tamil Nadu India delphine.sdc@saveetha.com; 3 Oman Dental College (ODC). Muscat, Sultanate of Oman. kavetisyan@staff.odc.edu.om Muscat Sultanate of Oman kavetisyan@staff.odc.edu.om; 4 Department of Prosthodontics, Faculty of Stomatology, Yerevan State Medical University after Mkhitar Heratsi. Yerevan, Armenia. heboyan.artak@gmail.com Yerevan State Medical University Department of Prosthodontics Faculty of Stomatology Yerevan State Medical University after Mkhitar Heratsi Yerevan Armenia heboyan.artak@gmail.com

**Keywords:** CAD-on crowns, BioHPP, PEKK, Finite element analysis, Stress distribution, deformation, coronas CAD, BioHPP, PEKK, análisis de elementos finitos, distribución de tensiones, deformación

## Abstract

**Objective:**

Zirconia-based restorations are widely used for fixed partial dentures but are prone to veneering porcelain chipping. The CAD-on technique, combining a zirconia or polymer coping with a lithium disilicate veneer, may enhance biomechanical performance. This study evaluated stress distribution and deformation of different CAD-on combinations versus conventional hand-layered zirconia crowns using finite element analysis (FEA).

**Materials and Methods:**

Mandibular molar crowns were modelled in four groups: Group 1 - conventional hand-layered zirconia; Group 2 - zirconia coping with lithium disilicate veneer; Group 3 - BioHPP (polyethyleetherketone) coping with lithium disilicate veneer; Group 4 - PEKK (polyethyleketoneketone) coping with lithium disilicate veneer. A 280 N static occlusal load was applied at five points of occlusal surface. Stress and deformation were analyzed with ANSYS; results were compared statistically (ANOVA, post hoc Tukey, p < 0.05).

**Results:**

Group 1 showed the highest occlusal von mises stress (1.341 ± 0.086) and deformation (0.099 - 0.100 mm). CAD-on crowns significantly reduced stress and deformation, with BioHPP and PEKK groups showing the most favorable outcomes.

**Conclusion:**

CAD-on crowns, especially with polymer copings, provided superior biomechanical performance compared to conventional hand-layered zirconia crowns.

**Clinical implementation:**

CAD-on crowns, particularly those with BioHPP and PEKK copings, demonstrated lower stress concentration and deformation, indicating their suitability for patients with high occlusal loads or parafunctional habits. Incorporating these polymer-based copings may improve long-term success rates compared to conventional hand-layered zirconia crowns.

## RESUMEN

**Objetivo:**

Las restauraciones con zirconio se utilizan ampliamente en prótesis parciales fijas, pero son propensas al astillamiento de la porcelana de recubrimiento. La técnica CAD-on, que combina una cofia de zirconio o polímero con una carilla de disilicato de litio, puede mejorar el rendimiento biomecánico. Este estudio evaluó la distribución de la tensión y la deformación de diferentes combinaciones de CAD-on respecto de coronas de zirconio convencionales estratificadas a mano mediante análisis de elementos finitos (FEA).

**Materiales y métodos:**

Las coronas molares mandibulares se modelaron en cuatro grupos: Grupo 1: zirconio convencional estratificado a mano; Grupo 2: cofia de zirconio con carilla de disilicato de litio; Grupo 3: cofia de BioHPP (polietilenetercetona) con carilla de disilicato de litio; Grupo 4: cofia de PEKK (polietilenecetonecetona) con carilla de disilicato de litio. Se aplicó una carga oclusal estática de 280 N en cinco puntos de la superficie oclusal. Se analizaron la tensión y la deformación con ANSYS. Los resultados se compararon estadísticamente (ANOVA, Tukey post hoc, p < 0,05).

**Resultados:**

El grupo 1 mostró la mayor tensión oclusal de von Mises (1,341 ± 0,086) y deformación (0,099 - 0,100 mm). Las coronas CAD-on redujeron significativamente la tensión y la deformación, y fueron los grupos BioHPP y PEKK los que mostraron los resultados más favorables.

**Conclusión:**

Las coronas CAD-on, especialmente con cofias de polímero, proporcionaron un rendimiento biomecánico superior en comparación con las coronas de zirconio convencionales estratificadas a mano.

**Implementación clínica:**

Las coronas CAD-on, en particular aquellas con cofias BioHPP y PEKK, mostraron una menor concentración de tensión y deformación, lo que indica su idoneidad para pacientes con altas cargas oclusales o hábitos parafuncionales. La incorporación de estas cofias de polímero puede mejorar las tasas de éxito a largo plazo en comparación con las coronas de zirconio convencionales estratificadas a mano.

## INTRODUCTION

Fixed partial dentures (FPDs) are widely regarded as a preferred treatment option for the replacement of missing teeth, largely due to their fixed nature within the oral cavity and their cost-effectiveness compared to dental implants ^(1, 2)^. Among the various materials employed in prosthodontics, ceramics have gained significant prominence, particularly in the fabrication of FPDs and other dental restorations ^(3, 4)^. Owing to their excellent esthetic qualities, ceramics play a central role in contemporary esthetic dentistry ^(5, 6)^. Commonly used ceramic systems include glass ceramics, alumina, and zirconia, which together constitute the primary choices for dental prostheses.It is challenging to achieve a good and predictable esthetic outcome for a hand layered crown as it is dependent on the technical skills of the dental technician. There is a need for a digital input software where software can produce good morphology of crowns which will be highly esthetic similar to natural morphology, thus reducing the dependency on human skill. Hence there is a need for a CAD on technique.

The clinical success of all-ceramic crowns and fixed partial dentures (FPDs) is largely dependent on the mechanical properties of their core materials. Yttria-stabilized tetragonal zirconia polycrystal (Y-TZP) is currently regarded as the core material of choice for posterior all-ceramic restorations, particularly FPDs, owing to its superior flexural strength and fracture toughness compared to alternative ceramic cores. Studies have consistently demonstrated that zirconia-based restorations exhibit higher flexural strength than other ceramic systems, such as lithium disilicate, regardless of the veneering technique used ^7^. Despite these advantages, the veneering porcelain chipping remains a frequent complication associated with zirconia-based restorations. Aboushelib et al. emphasized that the core-veneer interface represents one of the most vulnerable points in layered all-ceramic systems, significantly influencing their long-term performance ^8^. The IPS e.max® CAD-on system was developed to address this issue, utilizing lithium disilicate (IPS e.max® CAD; Ivoclar Vivadent) as the veneering material. Lithium disilicate has gained popularity in restorative dentistry due to its favorable esthetic properties and clinical durability, whether used as a core material or in monolithic form through CAD/CAM milling or pressing techniques. Although its flexural strength is lower than that of zirconia, long-term studies have reported promising outcomes, with only 2.5% chipping after four years ^(9, 10)^. In the CAD-on approach, both the Y-TZP core and lithium disilicate veneer are fabricated using CAD/CAM technology and subsequently joined with a fusion glass-ceramic (IPS e.max® CAD-Crystall./Connect; Ivoclar Vivadent), which transitions from solid to flowable under high-frequency vibration (Ivomix; Ivoclar Vivadent).

A pivotal study by Schmitter et al. (2012) compared conventional layering with the CAD-on method ^11^. Their findings revealed that 87.5% of restorations fabricated with conventional layering failed during in vitro aging prior to fracture testing, whereas CAD-on restorations not only withstood the aging process but also demonstrated fracture resistance values as high as 1600 N. The authors concluded that CAD/CAM lithium disilicate veneering over a Y-TZP framework offers a promising strategy to mitigate fatigue-related failures in all-ceramic restorations.

There are various methods for evaluating the stress on different CAD on materials. Finite element analysis is one of the methods used for evaluating the stress concentrations on different CAD on materials like zirconia, PEEK, BioHPP ^12^. The FEM evaluates the biomechanical impacts of different treatment modalities and serves as an approximation technique to depict both deformation and three-dimensional stress distribution in bodies subjected to stress ^13^. Zirconia restorations partially veneered with porcelain have demonstrated superior fatigue resistance compared to those fully coated with porcelain. However, the biomechanical advantages of extending porcelain coverage to the occlusal surface remain uncertain, as the literature provides inconclusive evidence on this aspect ^14^. The discrepancies observed between the two ceramic types are thought to be related to pre-existing flaws, leading to chipping or delamination. From a manufacturing perspective, computer-aided design/computer-aided manufacturing (CAD/CAM) technology has revolutionized the processing of high-strength ceramics, enabling efficient and precise fabrication ^15^. The use of CAD software not only facilitates the design of crowns with varied morphologies but also allows for the integration of biomechanically favorable concepts, tailored to the complexity of individual cases, thereby supporting more personalized treatment approaches ^16^^,^^17^. Although some studies have been done on the longevity of CAD on materials, there is limited evidence on the stress evaluation between different CAD on materials

The aim of the study is to check the stress evaluation between different CAD on materials over conventional layered zirconia. The null hypothesis was that there would be no difference in stress evaluation measured through FEA (finite element analysis) between different CAD on materials.

## MATERIALS AND METHODS

### Study Design

This is an in vitro study and approval by the university (SIMATS- Saveetha institute of Medical and Technical Sciences) ethics committee (SRB/SDC/PROSTHO-2302/25/115) was taken prior to starting the study. The following groups were used for the study- Group 1: Control (zirconia coping with hand layered ceramic); group 2: Zirconia Coping with lithium disilicate veneer; group 3: BioHPP coping with lithium disilicate veneer; group 4: PEKK coping with lithium disilicate veneer.

### Modelling and meshwork

First mandibular molars (typodont teeth) were prepared. Occlusal convergence was 12 degree and height were 5 mm. The veneer and corresponding tooth structures were scanned and the resulting geometries were exported in STL format. These STL files were subsequently cleaned and remeshed to ensure geometric fidelity and mesh quality (Nodes - 4664499, Elements - 2700186). Cement layers of specified thicknesses were then digitally modeled and accurately positioned to replicate the natural alignment of the veneer-tooth interface.

### Stress and deformation analysis

**Figure 1:**
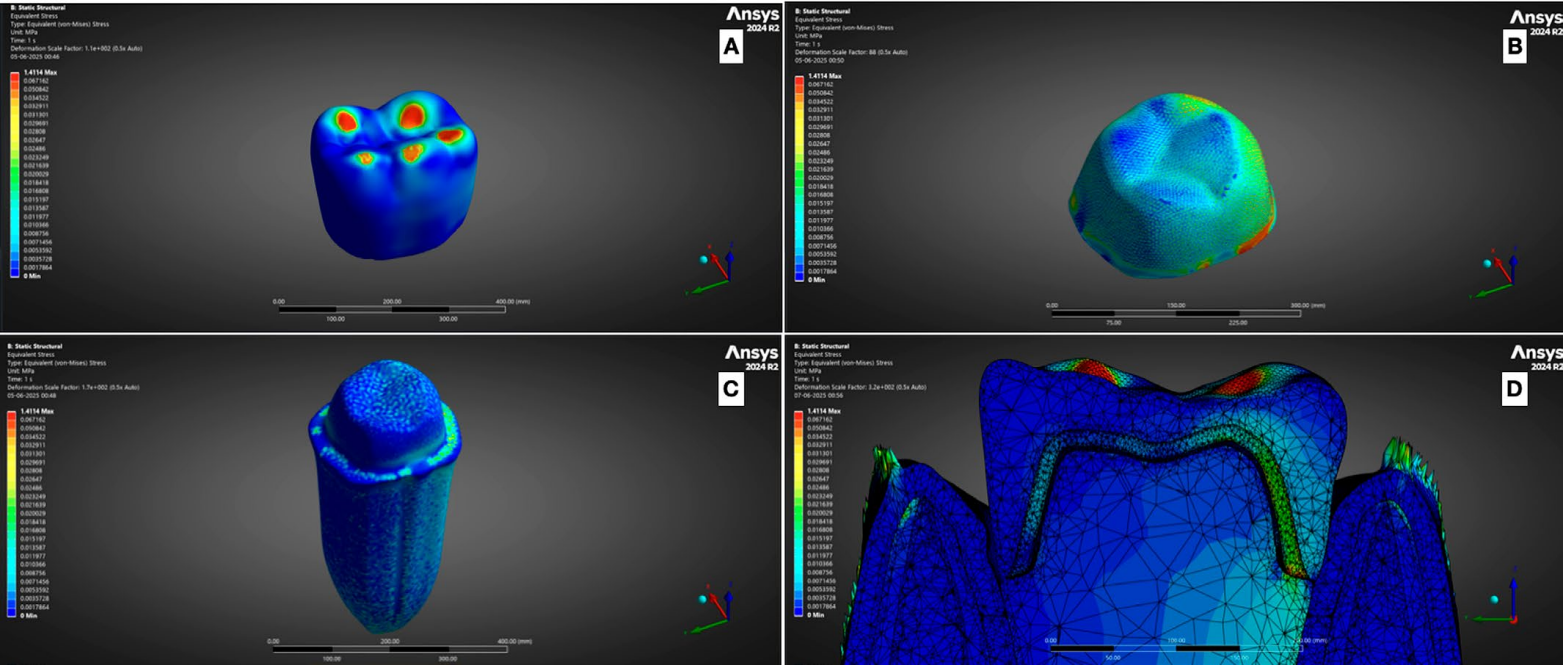
Von Mises stress on A. on veneer; B. On Coping; C. On abutment tooth; D. Overall view on mesh model

**Figure 2:**
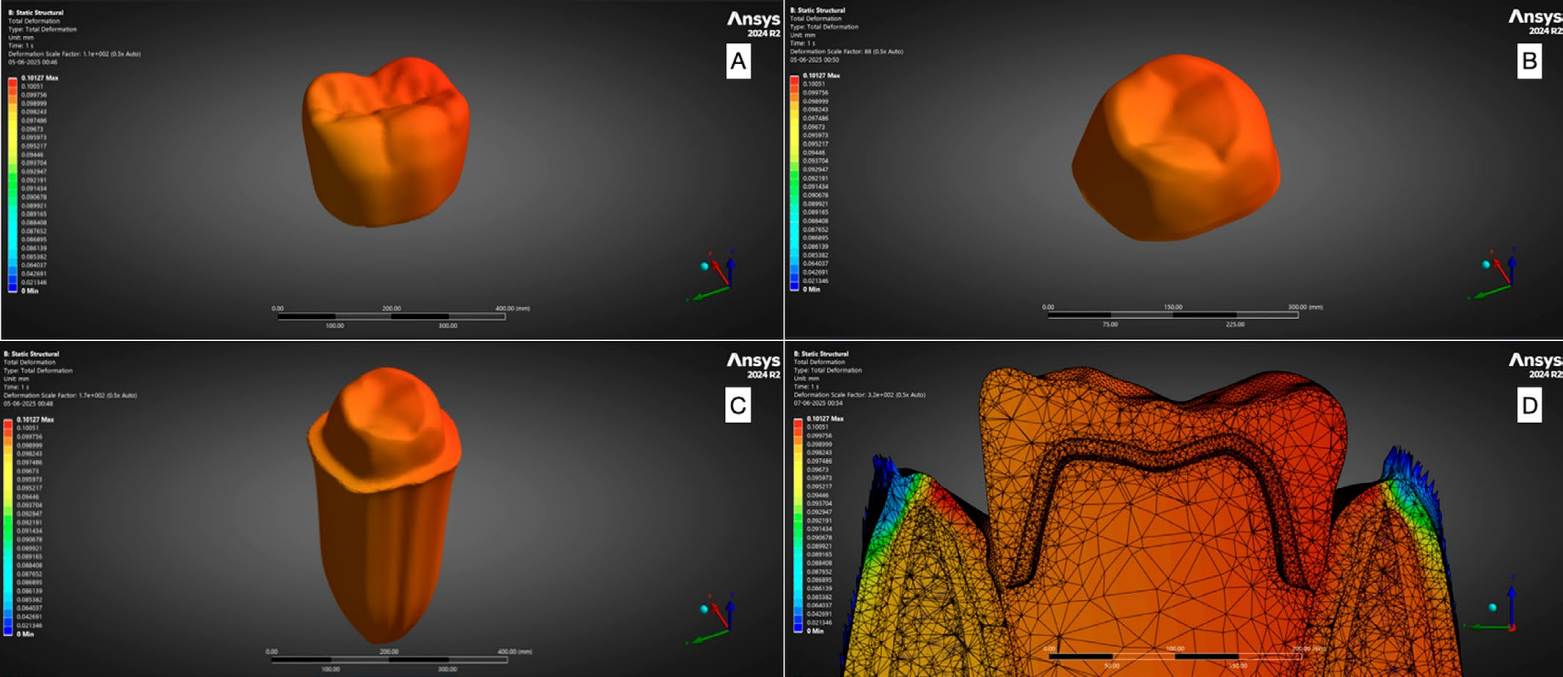
Deformation of A. veneer; B. Coping; C. Abutment tooth; D. Overall view on mesh model

The finalized STL models were converted into finite element (FE) meshes and imported into ANSYS (2024, r2), the selected finite element analysis (FEA) software. Appropriate boundary conditions, including fixed supports and loading regions, were defined at anatomically relevant domains within the FE model. Stress and deformation on maximum load were recorded from the software (Figures 1 and 2).

### Load Application

A static load of 280 N was applied at 90 degrees, perpendicular to five predetermined occlusal points to simulate functional masticatory forces. The mechanical performance of the system was evaluated using von Mises stress distribution and total deformation metrics, providing insight into the structural response under loading.

### Statistical analysis

The values were statistically analyzed using appropriate tests like One way ANOVA, Post Hoc Tukey's analysis. All statistical tests were done using SPSS (2023) software with a significant threshold set at p < 0.05. No evidence of ICC bias was observed in the analysis and interpretation.

## RESULTS

### Stress Distribution

The maximum von Mises stress analysis revealed variations among the groups where the group 1 (conventional hand layered zirconia) showed maximum occlusal von mises stress (1.411 MPa) (Table 1). Area wise Internal stress was assessed and the conventional hand layered zirconia crowns (Group 1) exhibited similar stress generation as they were statistically not significant (p>0.05) (Table 2).

**Table 1:** Pairwise comparison between groups 1,2,3 and 4 based on internal deformation

Groups	Maximum stress	Maximum deformation
Group 1: Control (zirconia coping with hand layered)	1.411	0.101
Group 2: Zirconia Coping with lithium disilicate veneer	0.404	0.081
Group 3: BioHPP coping with lithium disilicate veneer	0.186	0.080
Group 4: PEKK with lithium disilicate veneer	0.184	0.080

**Table 2:** Internal von mises stress comparison among groups on selected area (margin, coping and veneer)

Target	Groups	Mean ± SD	SE	95% CI		F-value	P-value
				Lower	Upper		
Margin	Group 1: Control (zirconia coping with hand layered)	0.002±0.002	0.001	0.001	0.006	0.061	0.970
	Group 2: Zirconia Coping with lithium disilicate veneer	0.003±0.004	0.002	0.003	0.106		
	Group 3: BioHPP coping with lithium disilicate veneer	0.003±0.002	0.001	0.000	0.066		
	Group 4: PEKK with lithium disilicate veneer	0.003±0.001	0.000	0.000	0.055		
Coping	Group 1: Control (zirconia coping with hand layered)	0.005±0.001	0.000	0.002	0.007	1.16	0.362
	Group 2: Zirconia Coping with lithium disilicate veneer	0.101±0.113	0.005	0.007	0.281		
	Group 3: BioHPP coping with lithium disilicate veneer	0.003±0.011	0.000	0.002	0.005		
	Group 4: PEKK with lithium disilicate veneer	0.003±0.000	0.000	0.002	0.003		
Veneer	Group 1: Control (zirconia coping with hand layered)	0.007±0.006	0.003	0.002	0.017	1.317	0.310
	Group 2: Zirconia Coping with lithium disilicate veneer	0.003±0.002	0.001	0.000	0.008		
	Group 3: BioHPP coping with lithium disilicate veneer	0.002±0.001	0.000	0.000	0.005		
	Group 4: PEKK with lithium disilicate veneer	0.004±0.002	0.001	0.000	0.008		
Abutment tooth	Group 1: Control (zirconia coping with hand layered)	0.003±0.002	0.001	0.000	0.007	0.72	0.558
	Group 2: Zirconia Coping with lithium disilicate veneer	0.003±0.001	0.000	0.001	0.004		
	Group 3: BioHPP coping with lithium disilicate veneer	0.004±0.002	0.001	0.000	0.008		
	Group 4: PEKK with lithium disilicate veneer	0.004±0.001	0.000	0.002	0.007		

### Deformation Analysis

Deformation values followed a similar trend. Group 1 (conventional hand layered zirconia) showed maximum deformation at occlusal surfaces (0.101) (Table 1). In contrast, CAD-on groups demonstrated significantly lower deformation values (p < 0.05). BioHPP and PEKK copings exhibited the most favorable results, with deformation values consistently lower than those observed with zirconia-based CAD-on copings (Table 3). For all the areas there was significant difference between group 1 (control) with any experimental cad on group (group 2, 3, 4) but no significant difference found between experimental cad on groups (Table 4).

**Table 3:** Internal deformation comparison among groups on selected area (margin, coping and veneer)

	Groups	Mean ± SD	SE	95% CI		F-value	P-value
				Lower	Upper		
Margin	Group 1: Control (zirconia coping with hand layered)	0.099±0.001	0.001	0.771	0.801	150.2	<0.001*
	Group 2: Zirconia Coping with lithium disilicate veneer	0.076±0.002	0.011	0.727	0.822		
	Group 3: BioHPP coping with lithium disilicate veneer	0.074±0.001	0.001	0.712	0.812		
	Group 4: PEKK with lithium disilicate veneer	0.076±0.002	0.001	0.728	0.824		
Coping	Group 1: Control (zirconia coping with hand layered)	0.985±0.001	0.000	0.09	0.101	292.16	<0.001*
	Group 2: Zirconia Coping with lithium disilicate veneer	0.757±0.001	0.000	0.73	0.77		
	Group 3: BioHPP coping with lithium disilicate veneer	0.752±0.001	0.000	0.73	0.77		
	Group 4: PEKK with lithium disilicate veneer	0.759±0.001	0.000	0.74	0.77		
Veneer	Group 1: Control (zirconia coping with hand layered)	0.099±0.000	0.000	0.098	0.099	256.6	<0.001*
	Group 2: Zirconia Coping with lithium disilicate veneer	0.075±0.001	0.000	0.72	0.79		
	Group 3: BioHPP coping with lithium disilicate veneer	0.075±0.001	0.000	0.72	0.79		
	Group 4: PEKK with lithium disilicate veneer	0.075±0.001	0.000	0.72	0.79		
Abutment tooth	Group 1: Control (zirconia coping with hand layered)	0.099±0.000	0.000	0.098	0.100	448.57	<0.001*
	Group 2: Zirconia Coping with lithium disilicate veneer	0.075±0.001	0.000	0.073	0.076		
	Group 3: BioHPP coping with lithium disilicate veneer	0.075±0.011	0.000	0.073	0.076		
	Group 4: PEKK with lithium disilicate veneer	0.075±0.001	0.000	0.072	0.076		

**Table 4:** Pairwise comparison between groups 1,2,3 and 4 based on deformation

parameter	Groups	Mean difference	SE	95%CI		P-value
				Lower	Upper	
Margin	Group1 vs Group 2	0.022	0.001	0.018	0.026	<0.001*
	Group 1 vs Group 3	0.024	0.001	0.020	0.028	<0.001*
	Group 1 vs Group 4	0.023	0.001	0.019	0.027	<0.001*
	Group 2 vs Group 3	0.002	0.001	0.001	0.004	0.387
	Group 2 vs Group 4	0.000	0.001	0.003	0.005	0.982
	Group 3vs Group4	0.001	0.001	0.005	0.006	0.588
Coping	Group1 vs Group 2	0.022	0.000	0.019	0.025	<0.001*
	Group 1 vs Group 3	0.023	0.000	0.020	0.026	<0.001*
	Group 1 vs Group 4	0.022	0.000	0.019	0.024	<0.001*
	Group 2 vs Group 3	0.000	0.000	0.002	0.003	0.928
	Group 2 vs Group 4	0.001	0.000	0.002	0.003	0.999
	Group 3vs Group4	0.000	0.000	0.003	0.005	0.879
Veneer	Group1 vs Group 2	0.024	0.001	0.020	0.027	<0.001*
	Group 1 vs Group 3	0.024	0.001	0.021	0.027	<0.001*
	Group 1 vs Group 4	0.023	0.001	0.020	0.026	<0.001*
	Group 2 vs Group 3	0.000	0.001	0.003	0.004	1.000
	Group 2 vs Group 4	0.000	0.001	0.003	0.005	0.995
	Group 3vs Group4	0.000	0.001	0.002	0.003	0.995
Abutment tooth	Group1 vs Group 2	0.024	0.000	0.021	0.026	<0.001*
	Group 1 vs Group 3	0.024	0.000	0.022	0.025	<0.001*
	Group 1 vs Group 4	0.025	0.000	0.021	0.026	<0.001*
	Group 2 vs Group 3	0.000	0.000	0.001	0.002	1.000
	Group 2 vs Group 4	0.000	0.000	0.002	0.003	0.989
	Group 3vs Group4	0.000	0.000	0.001	0.002	0.989

*P-value significant at 0.05, P-value was derived from Post Hoc Tukey test

## DISCUSSION

Finite element analysis (FEA) has proven to be an invaluable tool for investigating biomechanical performance of restorative materials and crown designs ^18^. In the present study, stress distribution and internal deformation patterns of different CAD-on material combinations were compared with the conventional layered zirconia crown. The results demonstrate that material selection significantly influences occlusal stress concentrations and deformation, thereby rejecting the null hypothesis that no difference would exist between groups. The superior biomechanical performance of BioHPP and PEKK copings observed in this study may be explained by their elastic modulus, which more closely approximates that of natural dentin compared to rigid zirconia. Zirconia, with its high stiffness, tends to concentrate stresses at the veneer-core interface, predisposing the restoration to localized stress peaks and subsequent veneer chipping. In contrast, BioHPP and PEKK, being high-performance polymers with dentin-analog mechanical behavior, act as effective stress absorbers, distributing occlusal loads more homogeneously across the crown structure. These findings are consistent with earlier reports by Schmitter et al. and Stawarczyk et al. ^11^, who emphasized the improved biomechanical compatibility and reduced veneer failures associated with polymer-based copings.Overall, both stress concentration and deformation analyses consistently indicated that CAD-on restorations provided superior biomechanical performance compared to conventional layered zirconia crowns.

The above findings are also consistent with previous studies indicating that hand-layered zirconia restorations are more prone to stress concentration and subsequent veneer chipping due to the heterogeneity of the veneering interface ^19^^,^^20^. The CAD-on technique, by contrast, ensures a uniform veneer-core adaptation via CAD/CAM milling and glass-ceramic fusion, which appears to distribute occlusal loads more evenly across the restoration ^21^. Interestingly, the internal stresses within the coping, veneer, and abutment tooth structures did not differ significantly between groups (p > 0.05), suggesting that the major biomechanical advantage of CAD-on systems lies in occlusal load management rather than in core or abutment stress shielding. The deformation analysis further substantiated these findings. The conventional layered zirconia group exhibited the greatest internal deformation across all components, particularly at the veneer (mean -0.099 ± 0.000) and occlusal surface (mean -0.1000 ± 0.000). In contrast, all CAD-on groups showed significantly lower deformation values, with BioHPP and PEKK copings again yielding the lowest deformation magnitudes. This reduction in deformation can be attributed to the intrinsic differences in elastic modulus between zirconia, high-performance polymers (PEKK, BioHPP), and lithium disilicate veneers. Zirconia, while highly rigid, creates stress concentration at the veneer interface due to mismatched stiffness, which predisposes restorations to chipping ^22^. Conversely, polymers such as PEKK and BioHPP possess lower elastic modulus closer to dentin, thereby acting as stress absorbers and reducing occlusal deformation. These findings align with prior reports suggesting that polymer-based copings enhance biomechanical compatibility and mitigate catastrophic veneer failures ^23^^-^^25^. From a clinical standpoint, the observed differences are highly relevant. The conventional hand layered zirconia crowns, despite their popularity, demonstrated higher susceptibility to stress concentration and deformation, which correlates with the high clinical incidence of veneer chipping and delamination reported in the literature ^26^^-^^28^. On the other hand, CAD-on systems, particularly those employing polymer-based copings offer a promising alternative by reducing stress peaks and improving load distribution. Lithium disilicate, used as a veneer in the CAD-on technique, provides favorable esthetics while maintaining adequate flexural strength. When fused to zirconia or polymer copings, it appears to overcome the limitations of conventional hand-layering and contributes to improved biomechanical reliability ^29^^,^^30^. Previous literature supports the Polymer for its high performance ability ^31^^-^^33^. The superior performance of PEKK and BioHPP in stress and deformation reduction may further support their use in patients with parafunctional habits or high occlusal loads. Schmitter et al. ^11^ previously demonstrated that CAD-on restorations exhibited significantly higher fracture resistance compared to layered zirconia restorations after in vitro aging. The present FEA findings are in agreement, showing lower stress concentrations and deformation in CAD-on systems. Additionally, earlier reports have highlighted the weak core-veneer bond strength in layered restorations as a primary cause of failure^34^, our study suggests that CAD-on systems may effectively overcome this drawback. Although few studies have examined PEKK and BioHPP in crown designs, their favorable performance in the present analysis suggests that they can be used as viable alternatives to zirconia due to their elastic modulus compatibility and reduced risk of catastrophic failure ^35^^-^^38^.

Despite providing valuable insights, this study has several limitations inherent to its in vitro FEA design. The analysis applied a static load, which does not replicate the dynamic, cyclic, and multi-directional nature of masticatory forces or the effects of fatigue over time. Factors such as the oral environment, thermal cycling, humidity, and the potential for degradation of the luting cement or materials were not simulated. The model assumed ideal geometries, perfect bonding interfaces, and isotropic material properties, which may not fully reflect clinical realities with inherent flaws and variations. Furthermore, the load was applied to a single crown on a prepared tooth, not accounting for the biomechanical complexities of a full dental arch or the periodontal ligament's shock-absorbing capacity. Therefore, while the results are indicative of comparative biomechanical performance, they should be validated through long-term in vitro fatigue testing and clinical studies.

## CONCLUSION

Conventional layered zirconia crowns demonstrated higher stress concentration and deformation compared to CAD-on restorations. Among CAD-on combinations, BioHPP and PEKK copings veneered with lithium disilicate exhibited the most favorable biomechanical performance by reducing occlusal stresses and internal deformation. These findings suggest that CAD-on systems, particularly those employing high-performance polymers, may provide enhanced durability and reduced risk of veneer chipping compared to traditional layered zirconia crowns. Further long-term in vitro and clinical studies are necessary to validate these advantages.
